# ReaLSAT, a global dataset of reservoir and lake surface area variations

**DOI:** 10.1038/s41597-022-01449-5

**Published:** 2022-06-21

**Authors:** Ankush Khandelwal, Anuj Karpatne, Praveen Ravirathinam, Rahul Ghosh, Zhihao Wei, Hilary A. Dugan, Paul C. Hanson, Vipin Kumar

**Affiliations:** 1 University of Minnesota, Department of Computer Science and Engineering, Minneapolis, 55455 USA; 2 Virginia Tech, Department of Computer Science, Blacksburg, 24060 USA; 3 Beijing University of Technology, Department of Information and Communication Engineering department, Beijing, 100124 China; 4 Center for Limnology, University of Wisconsin-Madison, Madison, WI 53706 USA

**Keywords:** Limnology, Hydrology, Water resources, Ecology

## Abstract

Lakes and reservoirs, as most humans experience and use them, are dynamic bodies of water, with surface extents that increase and decrease with seasonal precipitation patterns, long-term changes in climate, and human management decisions. This paper presents a new global dataset that contains the location and surface area variations of 681,137 lakes and reservoirs larger than 0.1 square kilometers (and south of 50 degree N) from 1984 to 2015, to enable the study of the impact of human actions and climate change on freshwater availability. Within its scope for size and region covered, this dataset is far more comprehensive than existing datasets such as HydroLakes. While HydroLAKES only provides a static shape, the proposed dataset also has a timeseries of surface area and a shapefile containing monthly shapes for each lake. The paper presents the development and evaluation of this dataset and highlights the utility of novel machine learning techniques in addressing the inherent challenges in transforming satellite imagery to dynamic global surface water maps.

## Abstract (summary)

**Table:** 

Measurement(s)	surface area of lakes and reservoirs
Technology Type(s)	unsupervised machine learning on satellite derived data

## Background & Summary

This paper presents a new dataset, Reservoir and Lake Surface Area Timeseries (ReaLSAT), that provides the location and monthly surface extents of 681,137 inland lakes and reservoirs (south of 50°N) at a global scale from 1984 to 2015. This dataset was produced by applying novel machine learning techniques to Earth Observation (EO) data to achieve a comprehensive catalog of the location and dynamic surface extent of inland waters. This dataset specifically focuses on lakes and reservoirs with size greater than 0.1 sq. km. in temperate and tropical latitudes. The Data Records section provides rationale for the spatial and temporal scope of this dataset.

Before expanding on the dataset, we first identify what is included in our definition of ‘lakes and reservoirs’. Often we think of inland waterbodies as lakes or rivers, but inland waterbodies can be considered any accumulation of water on Earth’s continents, and include canals, wetlands, and vernal pools. Limnologists, who study inland water, often differentiate *lotic*, or flowing waters, from *lentic*, or still waters. This is the dividing line between rivers and lakes, but the delineation is murky, as some riverine lakes resemble both. Take for instance, Lake Pepin, a natural lake in the Mississippi River. Any bystander would assume it was part of the river, and yet it is referred to as a lake because of the natural sediment dam at its outflow. We further tend to differentiate natural lakes from reservoirs, the latter being defined as an enlarged natural or artificial lake created using a dam. However, even here, the definition is muddled when natural lakes that have a dam used to control water levels. Often these waterbodies are still considered natural lakes. Furthermore, millions of small waterbodies, such as emphermal and agricultural ponds, are rarely categorized as either. Finally, waterbodies can be highly dynamic in their extent – or even in their existence – in response to, e.g., variations in local weather and hydrology and human influences that operate locally, such as the creation of farm ponds, or globally, such as global demands for water-based ecosystem services. While this diversity in both type and extent presents an obvious complication for enumerating waterbodies, it also presents an opportunity to better understand humanity’s evolving influence on Earth’s natural resources^1–4^. Our goal is to map the dynamics of a large swath of the world’s lakes and reservoirs, including small lentic waters such as agricultural ponds or tailings ponds. Here, we will refer to all lentic waters as ‘lakes’ to avoid misrepresenting lakes vs. reservoirs. Since humans interact with and manage lakes as discrete systems, knowing their temporal dynamics of water availability is essential for decision-making, and patterns of change in these lakes can be tied to both local freshwater conservation challenges and global biogeochemical cycling. Achieving our goal of building a global dataset of lakes and their dynamics has required the innovation of new machine learning approaches that meld knowledge of the physical dynamics of water bodies with satellite remote sensing products.

Recently, Landsat imagery has been leveraged to identify not only the location of the world’s waterbodies but also their sizes and dynamics. Previous efforts to map global inland waters fall into two categories: (1) vector-based mapping, which provides static polygons of the world’s lakes and lacks any temporal dynamics^5–10^, and (2) raster-based mapping, which divides the Earth’s surface into pixels and documents the change in the presence of water over time in those pixels^11–17^. Both these categories of approaches provide an incomplete view of the world’s lakes in that the former does not indicate the dynamic nature of surface water, and the latter tracks the presence of water at the level of pixels but fails to associate these pixels with lakes. Furthermore, pixel maps tend to suffer from a significant amount of missing data due to clouds and labeling errors, making it difficult to simply aggregate pixels in a lake to build a dataset of surface area changes in lakes. In addition, some efforts that combine both these aspects (i.e., provide surface water dynamics of individual water bodies) are either regional in scale^18–20^ or focus on certain types of water bodies such as reservoirs^21^.

To date, HydroLakes is the one of the most comprehensive database of static lake polygons, and Global Surface Water (GSW) dataset^14^ is the state-of-the-art for pixel-based mapping for surface water change over time. HydroLAKES is an aggregation of multiple datasets and contains 1.4 million waterbodies. As we describe later, within the spatial region covered by the ReaLSAT dataset, only a small fraction of waterbodies available in ReaLSAT are also present in HydroLAKES. GSW is provided by a collaboration between European Space Agency’s Joint Research Centre (JRC) and Google. It provides pixel maps of water presence available monthly at a 30-meter resolution, which have been used to map the occurrence and persistence of global surface water.

While informative, the major limitation of GSW dataset is that it does not provide a link between individual pixels and actual water bodies. In limnology, the origin/type of lake is a very important regulator of ecosystem dynamics. For instance, reservoirs will have faster water flow and lower residence time than natural lakes, and therefore nutrient and carbon processing rates will differ^22^; floodplain lakes may dry periodically, leading to the denudation of sediments and changing CO2 emissions^23^; and artificially constructed waterbodies will likely have much higher rates of nutrient loading and methane production than natural lakes^24^. Hence, aggregating pixels to the level of unique ecological entities (e.g., lakes, reservoirs, farm ponds etc.) provides critical information that is necessary for ecological analysis. Furthermore, those entrusted with enacting policies for water resource management (i.e., agencies, states, municipalities), need information presented as discrete waterbodies.

Though the GSW dataset is considered state-of-the-art gridded product, it requires extensive post-processing operations to analyze lake dynamics. First, polygons have to be constructed from pixel-based land/water masks, and then extents can be extracted for different lakes. Second, these extents cannot be directly used because they suffer from classification errors and large amounts of missing data. These issues make it challenging to extract robust surface extent dynamics of individual lakes at both intra-annual and inter-annual scales from the GSW dataset.

ReaLSAT is built off the GSW land/water classification maps^14,25^ and uses a novel physics-guided machine learning^26^ approach, ORBIT (Ordering Based Information Transfer), that exploits physical properties of lake dynamics to overcome the challenge of data quality while providing information as dynamic lake polygons instead of pixels^27–29^. ORBIT is based on two key ideas – (1) bathymetry (topography) constraints regulate the areal extent of lakes. For example, within a given lake comprising a single basin, a pixel at a higher elevation cannot be filled until all the lower elevation pixels are filled. (2) The relative bathymetry can be estimated using imperfect water extent maps if they are available for many timesteps. ORBIT provides a robust solution to both correct and impute missing labels and has been validated on 94 large global reservoirs using altimetry derived surface height data^28^. To evaluate the quality of surface extent maps generated by ORBIT for small to medium-sized lakes for which there is no altimetry data, we evaluated surface extent maps for a random selection of lakes in the ReaLSAT dataset using manually created reference extent maps. Overall, our goal in creating ReaLSAT is to produce a comprehensive data set of lake area extent across a large swath of the global terrestrial surface that can be used by wider research community to study the impact of human action and changing climate on freshwater availability.

Note that our primary contribution is in fixing these missing labels (and labeling errors) in the GSW dataset using the ORBIT approach. We used the GSW dataset as the starting point for our product, as it is the state-of-the-art pixel based label data set that is widely used by the community. Our rigorous comparative analysis is primarily meant to convince readers that ReaLSAT is not introducing spurious changes in the GSW based labels, and most of the changes made to the labels are indeed correct (as shown by our validation).

## Methods

This section describes the two main phases of the processing pipeline used to create high-quality surface area dynamics of individual lakes from erroneous and incomplete pixel-based maps available from the GSW dataset. The first phase created the database of static lake polygons, and the second phase generated monthly scale extent dynamics of individual lakes identified by the first phase.

### Phase 1: Static lake database generation

The generation of static lake database consists of the following steps:

#### Extraction of lake polygons

We used the GSW ‘occurrence’ layer to identify pixels globally that are part of any lake. Specifically, the ‘occurrence’ layer provides a number between 0 and 100 for each pixel, representing the percentage of months a pixel was observed as water. We binarized the layer by selecting pixels with a percentage value greater than 10. A threshold of 10% was chosen to reduce the chances of including spurious lakes in the database due to  potential errors in the GSW database. However, using a threshold to prune out some pixel also has some limitations. First, the threshold of 10% used to extract connected components might join multiple distinct lakes that happen to get connected for 10% (or greater) of timesteps. Similarly, a single lake with multiple parts connected by very narrow and shallow channels might get broken into multiple lakes. Second, lakes that exist for less than 10% of the study duration such as ephemeral or newly established are missing  from the database.

Once the binary layer was obtained by using the 10% threshold, we performed a connected component analysis and considered each contiguous set of pixels as a lake. If the GSW labels were perfect, this entire lake extent will be covered with water for at least 10% of the total duration. We refer to this lake extent at 10% threshold as the reference lake extent, and the number of pixels as the reference lake size. To ensure that the ORBIT framework has sufficient pixels to perform label correction and imputation, we only considered lakes with a size greater than 0.1 sq. km. (i.e., a lake with at least 100 contiguous pixels) as part of the dataset.

**Fig. 1:**
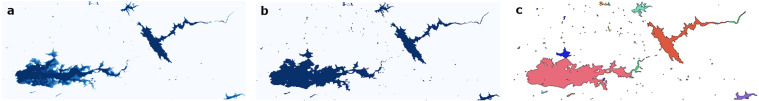
An illustrative example of lake polygon construction process. (**a**) GSW ‘occurrence’ layer. The colors from light blue to dark blue represents pixel value increases from 0 to 100. (**b**) The binary mask created by thresholding the occurrence layer at 10%. (**c**) Each connected component in the binary mask as shown in a unique color is identified as an individual lake.

Figure 1 illustrates the database generation process for a small region in the USA. Figure 1a shows the GSW ‘occurrence’ layer. The color scheme goes from light blue to dark blue as the ‘occurrence’ layer value increases from 0 to 100. Figure 1b shows the binary mask created by thresholding the ‘occurrence’ layer. Finally, this binary mask is used to extract individual connected components (sets of contiguous pixels) as shown in Fig. 1c. In this image, each connected component is shown in a different color and represents a unique lake in our database.

#### Identification and removal of river segments

Since GSW is a pixel-based dataset, the set of lakes extracted in the previous step also includes river segments. We used the geometric properties of lakes to remove river segments from the set of lake polygons and only focus on lakes and reservoirs. In particular, we calculated the number of erosion operations^30^ (referred to as *e*) needed to completely erode a lake with a reference size of N pixels. For narrow and long lakes (indicative of a river segment), it would take a small number of erosion operations to erode the lake, even if N is large. In particular, the lake cannot contain a square of size larger than (2*e* × 2*e*) because *e* erosion operations will remove this square. Hence, 4*e*^2^ represents the area of the largest square that can fit inside the lake, and the morphological score, $\frac{4{e}^{2}}{N}$, represents the fraction of the lake that is covered by the largest possible square. It is easy to see that lower values of this score would correspond to rivers, and larger values would correspond to square or globular-shaped lakes. A threshold of 0.05 was chosen for this score, which guarantees that at least 5% of the total lake exists as a perfect square. Even after thresholding based on this score, ReaLSAT still contains some lakes that appear to be river-shaped as no threshold would eliminate rivers without excluding too many lakes and reservoirs.

To provide additional information about presence of river segments in ReaLSAT, we overlaid a global river dataset (GRWL)^31^ and flagged all waterbodies in ReaLSAT that show overlap with rivers in this dataset. Specifically, we created a 4 km buffer as well as 1 km buffer around river lines in GRWL dataset. Upon close inspection, we found that a vast majority of these waterbodies are not river segments but include reservoirs on rivers and oxbow lakes around rivers.

### Phase 2: Surface extent dynamics generation

The second phase of our pipeline generated temporal dynamics of surface extent for all spatial polygons identified as lakes in the first phase. If the monthly scale pixel maps from the GSW dataset were accurate and complete, it would be straightforward to produce surface extent shapes at each timestep for individual lakes from these maps. However, as mentioned previously, these maps tend to suffer from large amounts of missing data and labeling errors. To address this, we use the ORBIT approach to correct GSW labels and impute missing labels using physics-based bathymetry constraints. Here, we describe the steps involved in this phase of the pipeline.

#### Extraction of initial surface extent maps

We extracted pixel-based water extent maps for each lake in the static lake polygon database at a monthly scale from GSW. Specifically, for a given lake, we created a bounding box around its static shape and extracted the monthly water extent maps. To avoid including other nearby lakes in the water extent maps, we only consider those water pixels connected to the target lake at least five times in the total time duration of 32 years. This step masks out pixels that may incorrectly connect the target lake with other nearby lakes. Each pixel in these water extent maps is labeled as either land, water, or missing. As mentioned earlier, these maps contain large amounts of missing labels and erroneous labels and thus require further processing to improve their quality.

#### Label correction and imputation using ORBIT

A novel machine learning approach, ORBIT (Ordering Based Information Transfer)^27–29^, was used to improve the quality of labels. ORBIT uses the inherent ordering constraint among pixels due to the bathymetry/elevation to detect physical inconsistencies in labels and impute missing labels. The elevation information introduces an inherent ordering in the locations, determining how a lake grows or shrinks. For example, assuming that a lake is composed of a single basin, if a pixel in the lake is filled with water, then all pixels in the lake with lower elevations should already be filled with water. If we had access to bathymetry data for every lake, we could use this elevation constraint to detect land/water labels that do not adhere to this physical constraint, and hence are likely erroneous. However, in reality, bathymetry information is available for relatively few lakes globally, and from *in-situ*, rather than remote sensing sources, limiting their utility for building a global scale product. In order to address this issue, the ORBIT approach uses an Expectation-Maximization framework^27^ that learns the elevation ordering implicitly from the multi-temporal water extent maps, which are then used to correct and impute missing labels. The ORBIT approach has been validated in the context of monitoring reservoirs using MODIS data^28^. In this paper, we further validate this approach using 2,095 water extent maps selected from ReaLSAT (see the Technical Validation section for more details).

**Fig. 2:**
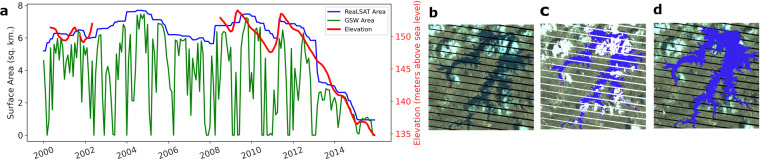
An illustrative example to demonstrate the utility of the ORBIT approach on the Araros reservoir in Brazil. (**a**) Comparison of GSW and ReaLSAT surface area timeseries with an altimetry-derived surface height timeseries. (**b**) LANDSAT-7 satellite image on November 2, 2012. (**c**) Water extent from GSW dataset (blue represents water, and white represents missing data). (**d**) Water extent map from ReaLSAT dataset.

Figure 2 illustrates the utility of the ORBIT approach using a reservoir in Brazil. Figure 2a compares the surface area timeseries from GSW (shown in green) and ReaLSAT (shown in blue) datasets. To calculate the area time series, we simply count the number of water pixels in GSW or ReaLSAT based water extent maps at each timestep. Since GSW has missing labels, the area timeseries shows very abrupt changes compared to the ReaLSAT timeseries. In order to validate these areal estimates, we compared them with variations in surface elevation of the reservoir obtained from an altimetry dataset (http://www.pecad.fas.usda.gov/cropexplorer/global_reservoir) (Birkett, 1995, Birkett and Beckley, 2010)^32,33^. Due to physical laws, surface area variations and surface elevation variations are monotonically related. Specifically, if the elevation increases, the area can either increase or stay the same but cannot decrease (and vice-versa). Hence, relative variations of surface elevation and surface area timeseries should be similar. As we can see, ReaLSAT based timeseries shows very good agreement with the elevation variations compared to GSW based timeseries. Figure 2b–d provides visual validation of the corrections and imputations made by the ORBIT approach for a single snapshot on November 2, 2012. As we can see, using the implicit bathymetry constraint, the ORBIT approach is able to successfully infer the labels on missing pixels.

#### Limitations of the ORBIT approach and potential solutions

The ORBIT approach makes two key assumptions, and its performance for a lake can be impacted if these two assumptions are violated. First, while correcting and imputing missing labels, it assumes that errors and missing data in the input water extent maps are not biased to either the water or land classes. In other words, it assumes that both water and land labels have a similar likelihood of being incorrect, and the occurrence of missing data is independent of land and water class (caused by unrelated factors such as clouds/shadows). In our analysis, we observed that the GSW dataset is more conservative in labeling a pixel as water. In other words, if a pixel is labeled as water in the GSW dataset, it has a higher probability of being true as compared to a land pixel. To address this issue, we introduced a slight modification to the original ORBIT approach by putting three times more penalty on updating a water pixel to land than changing a land pixel to water. Similarly, we observed that missing data are not independently distributed in some cases but are present around pixels where the GSW classification method was not confident about the label being land or water. Since ORBIT relies heavily on water extent maps in nearby months to fill missing data, it can introduce errors if the lake is changing considerably in nearby months (see the Technical Validation section for an illustration of this issue).

Second, the ORBIT approach assumes that a lake exists as a single basin, and its bathymetry can be estimated reliably from multi-temporal surface extent maps. However, in certain situations, this assumption does not hold. For example, if a lake disconnects into multiple sub-basins frequently, then the elevation order learned by ORBIT will be incorrect. Similarly, due to the proximity of the ponds used for rice (or fish) farming, adjacent ponds are often detected as a single lake. Since the filling and draining of these ponds do not follow any physical constraint (i.e., they can be filled in any order), the ORBIT approach can learn incorrect relative elevation ordering and introduce errors in extent maps for farm ponds. Furthermore, it can be challenging to estimate relative bathymetry for highly ephemeral lakes (i.e., lakes that exist for only a few timesteps). Finally, ORBIT assumes that the bathymetry does not change over time, which may not be accurate for certain types of lakes such as oxbow lakes and mining ponds.

To address some of these issues, we provide two quality flags that capture some of these aspects:

- *Connected Component Score* (*CS*): This score aims to detect if a lake exists as a set of separate components, indicating the violation of the topographic constraint. In particular, for any given timestep, we calculated the number of pixels that do not belong to the largest component as a penalty for that timestep. To calculate a single value for a lake, we aggregate the penalties across all timesteps as follows:
  1 $$CS=\frac{{\sum }_{t=1}^{T}P[t]}{{\sum }_{t=1}^{T}W[t]}$$
  where, *P*[*t*] is the penalty at timestep *t* and *W*[*t*] is the total number of water pixels at timestep *t*. An ideal lake will have a *CS* score of 0, and a highly disconnected lake will have a *CS* score close to 1.
- *Ephemeral Score* (*ES*): Highly ephemeral lakes that appear for a tiny amount of time do not have enough data to learn a robust elevation ordering, leading to incorrect areal estimates by the ORBIT approach. To capture the transient nature of lakes, we used a simple score that counts the number of timesteps a lake is less than 10% of its reference size. A very stable lake will have a score of 0.

With these scores, we can identify and filter lakes that are highly ephemeral or have multiple basins, where the label corrections and imputations of our ORBIT approach may have inaccuracies. However, beyond reporting these scores, we do not provide detailed categorization of the different types of identified lakes where topographic constraints are violated, such as farm ponds, mining lakes, and wetlands. A more detailed study on how to characterize different types of waterbodies using automated approaches can be developed as a follow-up to our current work.

## Data Records

ReaLSAT provides spatially explicit information on surface area variations of lakes. The static lake polygon dataset contains 681,137 individual lakes. A base shapefile provides the reference shape of these lakes. For each lake in the dataset, its monthly surface area timeseries is provided in a csv file, and its monthly shapes are provided as polygon shapefiles (.shp). The area values were calculated by aggregating 30 m × 30 m LANDSAT pixels (one pixel corresponds to 0.0009 square kilometers). All data records are available for download from Zenodo^34^ and a zoomable visualization is available at http://umnlcc.cs.umn.edu/realsat.

### Spatial and temporal scope

As highlighted previously, the dataset contains lakes above 0.1 square kilometers. Due to high computational time for large lakes, we provide surface area dynamics of 1120 lakes above 100 sq. km. at 300 m spatial resolution. Furthermore, the dataset only contains lakes between 50 N and 60 S. The limit of 50 N was chosen because a vast majority of lakes in the arctic region show inter-connectivity via narrow channels. Since ReaLSAT uses an automated approach, these lakes would often get disconnected because narrow channels are often not visible in the data. Furthermore, ORBIT assumes that lakes exist as a single bowl and hence these disconnected lakes will not satisfy the requirements of the framework. Therefore, we decided to exclude these lakes from the current version of the dataset because automated extraction of water bodies is not reliable in this region.

Even though lake extents are available at monthly scale, there are significant data gaps in major parts of the world before 2000 which is also true for the original GSW dataset. Furthermore, for waterbodies in tropical regions, there could be situations where data for consecutive months is missing. While the ORBIT approach can fill these gaps, the quality of imputations degrades as the amount of consecutive missing data increases. In the dataset, each monthly extent shape contains metadata information about % pixels that were imputed and corrected using the ORBIT approach. We also provide a code snippet that uses this metadata information to identify consecutive timesteps with large amounts of missing data, which can then be ignored during analysis. Finally, while the GSW dataset has been recently updated to 2020, it was only available until 2015 when we started this study. Hence, the temporal coverage of our dataset is between 1984 and 2015. The dataset will be extended to recent years in future updates.

### Quality

Since the temporal dynamics produced by the ORBIT approach are not reliable for lakes that violate its bathymetry constraints, we provide two quality flags for filtering reliable lakes: Ephemeral Score (ES) and Connected Component Score (CS). Specifically, we visually inspected lakes by ranking them according to these two scores and found the following thresholds on these scores to filter reliable lakes effectively: *ES* ≤ 156 & *CS* < 0.2. This results in a set of 462,574 lakes with reliable surface extent dynamics. However, these thresholds are not absolute, and we encourage the users to adopt different score thresholds as per their requirements in a region of study. These scores, along with the morphological score (used to remove river segments), are provided for all 681,137 lakes within the base shapefile.

Along with the dataset, we also provide all the reference information used in the evaluation of ReaLSAT dataset (as described in the technical validation section).

#### Overview of ReaLSAT

In this section, we provide a summary of the patterns observed in the ReaLSAT dataset.

##### Spatial coverage

**Fig. 3:**
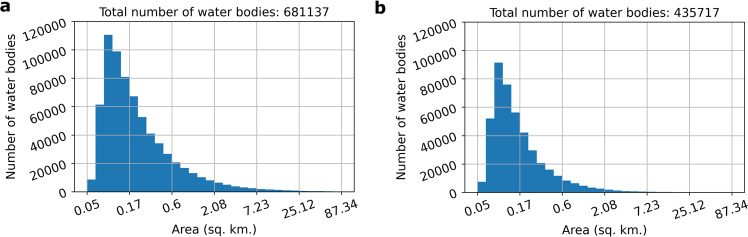
(**a**) Size distribution of lakes in ReaLSAT. (**b**) lakes included in ReaLSAT, but not present in HydroLAKES. For both figures, the x-axis was truncated to show lakes smaller than 100 sq. km. to avoid skewing of the axis by a few large lakes.

**Fig. 4:**
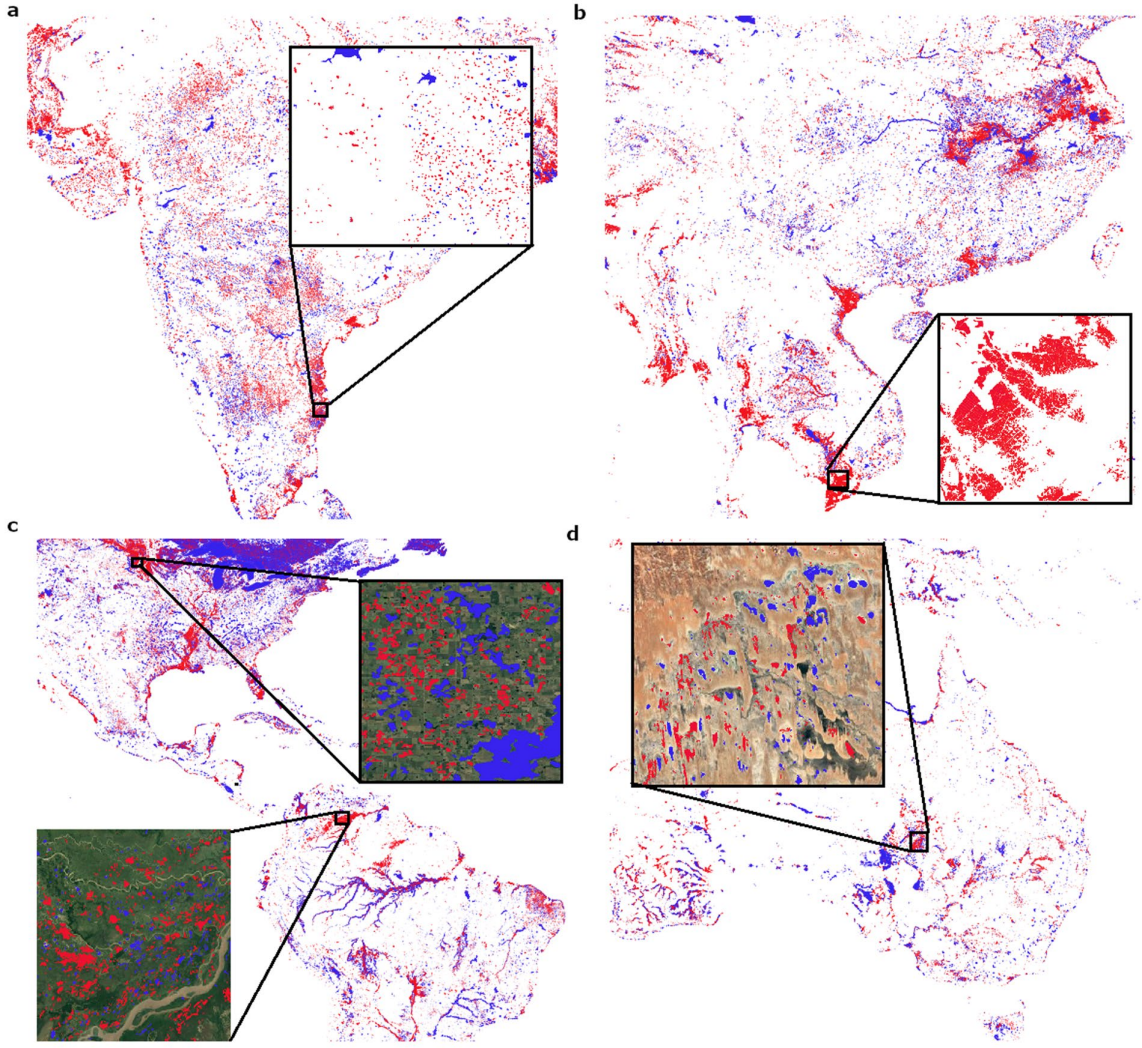
ReaLSAT provides comprehensive identification of surface lakes. Here, four regions are shown to highlight the lakes identified by ReaLSAT and HydroLakes (blue), as well as the lakes present in ReaLSAT but not HydroLakes (red). (**a**) Small reservoirs in India, (**b**) Water intensive agriculture in Vietnam, (**c**) Natural lakes in USA (top right), and wetlands in Venezuela (bottom left), and (**d**) Shallow lakes in Australia.

The size distribution of ReaLSAT lakes follows a power-law distribution, similar to the known distribution of all but the smallest lakes on Earth^35^ (Fig. 3a). Since ReaLSAT uses an automated process to identify lakes based on satellite imagery analysis, it can provide much more extensive coverage of inland waters than existing datasets such as HydroLAKES (a widely used dataset by the hydrology community), which are aggregations of multiple datasets produced by the community for different limnological use-cases over the years. In particular, since ReaLSAT is designed to automatically detect all types of lentic waters without any expert curation, it is beneficial for detecting waterbodies that have been traditionally overlooked in existing datasets such as farm ponds, cranberry bogs, rice paddies, or tailings ponds. For example, we found that out of the 681,137 lakes in ReaLSAT, 435,717 lakes are not present in the HydroLAKES dataset^6^. Fig. 3b shows the size distribution of the lakes that are missing from the HydroLAKES dataset. The size distribution also follows a power law, suggesting that lakes of all sizes are missing from HydroLAKES. Figure 4 shows a selection of some of the spatial clusters of the lakes that are missing from HydroLAKES. As illustrated through these regional examples, a wide variety of lakes and reservoirs in ReaLSAT are missing from HydroLAKES. Note that in Fig. 4c, a river-like pattern is forming around the Amazon river which might suggest that river segments are being included in the dataset. However, those lakes correspond to oxbow lakes around the river and not the river itself.

##### Temporal dynamics

Robust surface extent maps at a monthly scale enable an analysis of surface area changes in individual waterbodies. Supplementary Fig. S1 shows the aggregate surface area timeseries of 462,574 lakes (out of 681,137) that were flagged as reliable. Note that the timeseries before the year 2000 is shown in gray to signify that before 2000, the GSW dataset contains a lot of missing data in significant parts of the world (due to gaps in the LANDSAT data collection), and hence, the timeseries signal before 2000 should be interpreted with caution.

As shown in Supplementary Figs. S3 and S5, this dynamics can differ greatly for small (less than 100 sq. kms.) and large lakes (greater than 100 sq. kms.). Specifically, large lakes have shown a steady decline in total area since 2000s in each continent, where the pattern of change in the total area of smaller water bodies is less consistent over time and across continents. One common pattern for both small and large lakes is that the total surface water extent showed sudden increase in 2011 and the total extent was among the smallest in 2009. These relative magnitudes likely correspond to global precipitation and temperature changes in our study area.

Supplementary Figs. S2, S4 and S6 shows the total area variations for each continent separately for all, small and large lakes respectively. As we can see, all continents have very different area variations over the study period. South America has shown a very long drought pattern from 2002 till 2010. In contrast, Africa has shown a gradual increase in the total surface area among small lakes during this period (partially due to the addition of new reservoirs). Area variations in North America were consistent except for the year 2012. Australia had a significant increase in surface water for the period 2010–2012 for both small and large lakes.

## Technical Validation

In this section, we provide quantitative evaluation for both spatial coverage and temporal dynamics of ReaLSAT dataset.

### Spatial coverage

Since the dataset was created using satellite imagery analysis, it can provide more comprehensive coverage than existing datasets. However, using an automated process also has its challenges. It can invariably lead to the detection of spurious waterbodies because of issues in data (e.g., due to errors in GSW maps used as inputs in ReaLSAT).

**Fig. 5:**
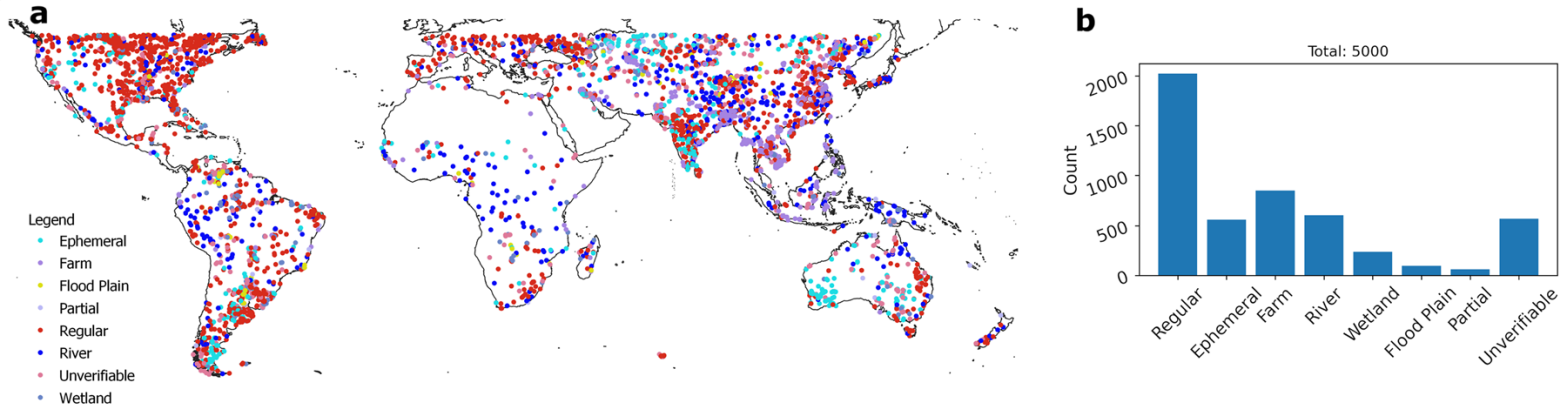
(**a**) Geographic location of 5000 randomly selected lakes used for manual evaluation of lake type. (**b**) Allocation of the 5000 manually referenced lakes to specific lake types. Regular implies a traditional lake or reservoir. Unverifiable implies that the lake type could not be identified based on the available Google Earth imagery.

To provide more insights into the types of lakes and potential issues in the spatial coverage of ReaLSAT, we randomly sampled 5,000 lakes out of 435,717 that are only present in ReaLSAT (i.e., not available in the HydroLAKES dataset). A human annotator used Google’s satellite imagery base layer to categorize these lakes. Figure 5a shows the geographical distribution of these lakes, and Fig. 5b shows the distribution of different lake types in the sample set. Out of the 5,000 lakes, the human annotator identified 2,019 traditional lakes and reservoirs where sufficient water was visible in the satellite imagery. Another 551 lakes in the sample set showed signs of a bowl-like depression but with no (or very little) water visible in the satellite imagery and were labeled as ephemeral. There were 861 other lakes that were tagged as farm ponds because they showed geometric patterns of farming in the imagery. This diversity of waterbody types discovered by ReaLSAT that were previously unreported by HydroLakes highlights one of the strengths of our approach. In limnology, the origin/type of lake is a very important regulator of ecosystem dynamics. For instance, reservoirs will have faster water flow/lower residence time than natural lakes, and therefore nutrient and carbon processing rates will differ; floodplain lakes may dry periodically, leading to the denudation of sediments; and farm ponds will likely have much higher rates of nutrient loading and methane production than non-agriculturally influenced lakes. Hence, capturing a more comprehensive range of waterbody categories can enable various scientific studies where knowing the origin/lake type could provide a critical understanding of the process.

Along with the lentic water types discovered in the sampled set, we also found that ReaLSAT identified 603 river segments missed by our morphological score filter. As stated earlier, this is an inherent challenge with automated approaches that use a fixed score threshold for eliminating river segments. Another 239 lakes were tagged as wetlands because of significant vegetation inside and around the lake polygon. There were also 97 lakes that were adjacent to rivers, which were labeled as riverine or floodplain lakes that were formed as a result of river channels meandering over time. Furthermore, there were 59 lakes where the polygons represented only a small portion of a larger lake and were labeled as partial. Finally, for 571 polygons, there was not enough evidence to tag them in any of the above categories. Since Google imagery represents only a single snapshot in time, these 571 waterbodies could not be definitively labeled as spurious (hence, they were labeled as unverifiable), highlighting a limitation of this evaluation pipeline. In particular, a vast majority of these waterbodies appear to be ephemeral based on their surface area timeseries (completely dry for extended periods of time). Hence, if the satellite imagery layer is from one of these timesteps, the annotator would not be able to confirm the presence of the lake.

To assess whether we would obtain a similar distribution of different waterbody categories in existing datasets, we performed a similar evaluation on another 5,000 lakes sampled from ReaLSAT where each polygon has some overlap (greater than 1 pixel) with a polygon from HydroLAKES. In this sampled set, the annotator identified 4,030 lakes as traditional lakes or reservoirs, 370 as ephemeral, 138 as farm ponds, 6 as river segments, 66 as wetlands, 95 as riverine or floodplain lakes, 20 as partial, and 275 as unverifiable.

Compared to previous distribution, this set of 5,000 waterbodies contains relatively fewer river segments and wetlands polygons in HydroLAKES, because these categories were manually identified and removed during HydroLAKES database creation^6^. Similary, this set contains relatively few farm ponds because HydroLAKES was created by manual curation of existing static databases and hence does not contain new farm ponds that got created over the years.

### Temporal dynamics

To assess the quality of surface extent maps, we performed a quantitative evaluation on a random selection of extent maps. These extent maps were compared against reference maps created by a human annotator using a semi-automated pixel classification procedure. This strategy of creating reference maps is used extensively in the remote sensing literature (e.g. see^36–39^). Next, we describe our evaluation process in detail.

#### Sample selection

**Fig. 6:**
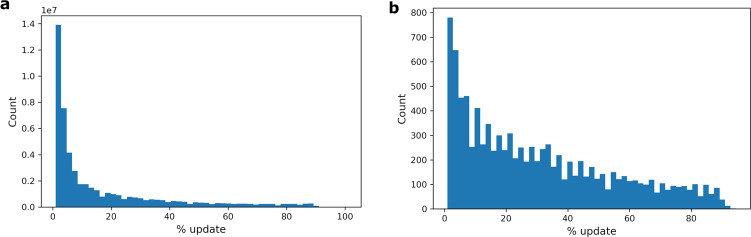
Distribution of updates made by the ORBIT approach. (**a**) distribution using candidate water extents (**b**) distribution using randomly selected 10000 water extent maps for evaluation.

There are 462,574 lakes out of 681,137 total lakes where the label updates (corrections and imputations) by the ORBIT approach have trust scores within our chosen thresholds (as described in the methods section). To evaluate these candidate lakes effectively, we focus on lake extent maps where the ORBIT approach resulted in a different map than the underlying GSW extent based map. Hence, we remove maps where no updates were made by the ORBIT approach (neither corrections nor imputations) from the candidate pool of extent maps used for evaluation. We also remove maps where the percentage of missing labels was more than 90% because these maps tend to suffer from significant cloud coverage. Hence, it would be challenging to generate reference maps. Since the GSW dataset has a significant amount of missing data for most places in the world before 2000, we evaluated maps only from 2000 onwards. These three filters left us with a total of 51,077,278 water extent maps considered for selection. Figure 6a shows the distribution of percentage pixels updated made by the ORBIT approach in these water extent maps. To evaluate the robustness of our approach in comparison to GSW maps, we randomly selected 10,000 water extent maps such that extents with significant updates are given higher weight to reduce the skew in distribution towards extents with relative less updates (Fig. 6b).

#### Sample pruning

**Fig. 7:**
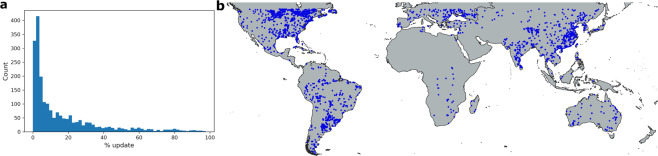
Summary of the dataset used for evaluating water extent maps. (**a**) Distribution of updates made by the ORBIT approach in the water extent maps selected for evaluation. (**b**) Geographical location of the lakes in the evaluation set.

From these randomly selected water extent maps, we removed maps for which a reference map could not be generated due to clouds or the inability of the annotator to distinguish between land and water. A final set of 2,095 water extent maps were considered for evaluation. Figure 7a shows the distribution of percentage updates in the final set of evaluation extents and Fig. 7b shows the geographical distribution of these extent maps.

#### Reference map generation

For these water extent maps, we created ground truth reference maps using a semi-automatic labeling process^37–39^. Specifically, the annotator selects land and water samples to train an SVM (Support Vector Machine) classification model for each image. The annotator keeps adding samples until a stable map is generated. As a final step, the annotator masks out pixels affected by clouds, cloud shadows, and any other region where the annotator is not confident about the accuracy of the reference labels. This process enables a quick and robust generation of reference maps. Supplementary Fig. S7 shows one of the reference maps in the evaluation set. While this strategy of comparing maps is different from the traditional approach of comparing pixels (often selected using stratified sampling), it provides a much more exhaustive evaluation of surface extent maps. The reference maps used for evaluation in this study are also available as part of the dataset.

#### Comparison

To compare the extent maps generated by ReaLSAT with the reference maps, we used accuracy as the evaluation metric, a widely used metric to measure the quality of classification maps. Accuracy is simply defined as the ratio of pixels with correct labels over a total number of pixels. Specifically, we assign 1 to water pixels and 0 to land pixels. Since GSW based extent maps contain missing labels, they are assigned a value of 0.5 to reflect the uncertainty between land and water. Accuracy is then calculated as follows:

2 $$Accuracy=1-\frac{1}{R\ast C}\mathop{\sum }\limits_{i=1}^{R}\mathop{\sum }\limits_{j=1}^{C}\left|ReferenceMap[i,j]-PredictedMap[i,j]\right|$$

where, *R* is the number of rows and C is the number of columns of the map.

**Fig. 8:**
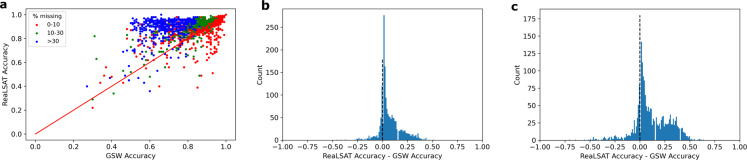
Comparison of accuracy values using GSW labels vs ReaLSAT labels. (**a**) Scatter plot of accuracy values using GSW labels vs ReaLSAT labels. (**b**) Histogram of difference in accuracy between ReaLSAT labels vs GSW labels. Positive value represents cases where ReaLSAT labels were more accurate than GSW labels. (**c**) Histogram of difference in accuracy values for the scenario where pixels labelled as land by both products as well as ground truth were removed to reduce the skew of surrounding land pixel on the accuracy values.

When the accuracy of RealSAT and GSW labels are compared, a vast majority of points lie above the diagonal 1:1 line, which implies that ReaLSAT labels were more accurate overall (Fig. 8a). In Fig. 8 the points are colored based on % of pixels where GSW labels were missing. To better show the improvement in RealSAT labeling, we plot the distribution of the difference in accuracy values between the two datasets as shown in Fig. 8b. A positive value indicates that the surface extent map from the ReaLSAT dataset had better accuracy than the map from the GSW dataset and vice versa. For ease of visualization, we plot this distribution after excluding cases where the accuracy from both datasets was equal. The positively skewed distribution demonstrates the efficacy of the ORBIT approach.

Note that the shape of a lake will influence the number of land pixels surrounding it, which might bias the accuracy values. For example, the reference map shown in Supplementary Fig. S7 contains more than 70% of land pixels. To address this bias, we also calculated accuracy values after removing pixels that were labeled as land by both datasets as well as the ground truth. This variation allows a more strict evaluation of water extent maps. Figure 8c shows the distribution of the difference in accuracy values under this scenario (after excluding cases with equal accuracy). As shown, a vast majority of the distribution is still towards positive values. Furthermore, the distribution has a larger spread towards high positive values, suggesting significant improvement made by the ORBIT approach.

From Fig. 8, we can see that for some cases ReaLSAT based extent maps are less accurate relative to GSW. As described earlier, violation of assumptions made by the ORBIT approach could lead to the observed poor performance. Out of 2,095 extent maps, GSW labels show better accuracy than ReaLSAT for 323 of them. On visual analysis of errors in these maps, we found that 165 maps are slightly different only at the lake’s boundary. We categorized the remaining extent maps based on the reason behind the observed poor performance. In particular, 45 maps have poor performance due to occlusion of water surface by algae, 18 maps contain farm ponds, 8 contain mining lakes, 27 maps have unreliable bathymetry, 30 maps have issues due to the weighting factor used by ORBIT approach, and 30 maps have class conditional missing data. All the reference maps and corresponding maps from GSW and ReaLSAT are provided with the dataset.

Next, we describe some of these cases in detail.

**Impact of algae:** It can be difficult to visually differentiate surface algae or floating aquatic plants from terrestrial vegetation^40^, as they have similar reflectance spectra. Therefore, surface algal blooms often get incorrectly labeled as land in the reference maps. However, in most cases, the appearance and disappearance of algae on a lake are independent of the bathymetry. Thus, algae pixels get detected as physically inconsistent by the ORBIT approach, and consequently, these pixels are updated based on the labels of other pixels without algae. In many cases, while the accuracy with respect to the reference map is poor (because algae get labeled as land), ReaLSAT based extent maps are closer to the true extent of the lake. For example, Supplementary Fig. S8 illustrates the impact of algae on the extent mapping of Center Lake, Texas. In this example, the bimodal distribution of fraction values (either low or high) reveals high confidence in lake persistence (Supplementary Fig. S8b). On Oct 22, 2008, false-color composite processing of LANDSAT-5 imagery reveals a strong vegetative signal on the west side of the lake (Supplementary Fig. S8c). Since we know that this is a lake, we can assume that the west side of the lake is experiencing a large surface algal bloom with a similar reflectance to the surrounding terrestrial landscape. Because of the strong vegetative reflectance signal, the semi-automated reference mapping labels the west side of the lake as land (Supplementary Fig. S8d), as does most GSW labels (Supplementary Fig. S8e). Conversely, the ReaLSAT extent map labels the west side of the lake as water (Supplementary Fig. S8f). However, we calculate accuracy based on the semi-automated reference map (Supplementary Fig. S8d). Due to this, the GSW extent map is considered more accurate than the ReaLSAT map, even though this is not true because the reference map is incorrectly labeled. Therefore, some negative accuracy values may be a misrepresentation of reality due to surface algal blooms.

**Impact of variable bathymetry:** Even though we tried to remove lakes with unreliable bathymetry by using score-based filters defined in an earlier section, not all cases were removed. For example, agricultural ponds often have small sections that are connected and change shape based on agricultural needs. Supplementary Fig. S9 highlights an example of labeling issues on agricultural ponds in Mexico. In this area, satellite imagery and the GSW fraction map confirm the presence of agricultural ponds (Supplementary Fig. S9a,b). These individual ponds are filled and drained based on operational decisions and do not follow a consistent pattern of growing or shrinking. Thus, the ORBIT approach can introduce spurious updates in water extent maps for these farms. In the Landsat-5 imagery from 2009–10–08, some of the ponds are dry, while others are filled (Supplementary Fig. S9c). This distribution of water is evident from a visual inspection and is confirmed in the semi-automated reference map (Supplementary Fig. S9d). Due to the similar elevations between the individual pond sections, the ORBIT approach spuriously fills the remaining sections with water based on the incorrectly learned bathymetry (Supplementary Fig. S9f). While quantification of such uncertainties is outside the scope of this paper, we hope that the wider research community can use RealSAT to address such questions. In particular, changes in bathymetry of a lake can be identified using spatial-temporal patterns in the label corrections. Specifically, if the elevation of some pixels in a lake increases after a certain time (e.g., sediment deposits leading to increase the elevation of a pixel), they will appear as physically inconsistent to the ORBIT framework, and hence the labels for these locations will be changed from land to water much more frequently after this increase in elevation.

**Impact of bias in errors and missing data:** As mentioned earlier in the methods section, based on our observation, the confidence of water labels is higher than land labels in the GSW dataset. To account for this bias, we used a weighting factor of 3 for the water class. While this weighting factor improves the ORBIT approach’s performance in most cases, this assumption leads to an overestimation of water for some lakes. For example, Supplementary Fig. S10 compares the water extent maps with and without the weighting factor for a small reservoir in eastern Brazil. As we can see, the GSW labels contain false positives, and due to the weighting factor of 3, ORBIT prefers to update the land labels to water which further increases the number of false positives, as shown in Supplementary Fig. S10e. However, if we use a weighting factor of 1 for this example, the ORBIT approach can effectively remove many of the false positives in the GSW map, as shown in Supplementary Fig. S10f.

Similarly, apart from missing data due to clouds in the GSW dataset, there can also be missing values on pixels where the GSW classification model is not confident. Hence, for some water extent maps, class-dependent missing data (compared to missing data which is class independent) adversely impact the ORBIT approach. For example, Supplementary Fig. S11 shows a water extent map for Zhongleng Reservoir in China, where missing data along the eastern edges is not independent but has resulted from ambiguous pixels around the lake where the GSW’s approach was not confident. In such a scenario, the ORBIT approach heavily relies on information from nearby timesteps to infer labels for missing pixels, leading to errors in ReaLSAT maps if there is a significant variation in lake extent in nearby timesteps, as shown in Supplementary Fig. S11e.

## Usage Notes

The dataset repository contains a Python based Jupyter notebook that provide additional details about all the data records and the code to visualize the data.

## Supplementary information

Supplementary: ReaLSAT, a global dataset of reservoir and lake surface area variations

## Data Availability

The data repository contains a jupyter notebook that provides the code to access the input GSW dataset and process it using the ORBIT framework.
