# Supplementary material for: ReaLSAT, a global dataset of reservoir and lake surface area variations
Source: Sci Data. 2022 Jun 21;9:356. doi: 10.1038/s41597-022-01449-5 (PMC9213522; doi:10.1038/s41597-022-01449-5)
Supplement: Supplementary file 1 — Supplementary: ReaLSAT, a global dataset of reservoir and lake surface area variations [file 41597_2022_1449_MOESM1_ESM.pdf]

Supplementary: ReaLSAT, a global dataset of reservoir and lake surface area variations

Ankush Khandelwal[1,*], Anuj Karpatne[2], Praveen Ravirathinam[1], Rahul Ghosh[1], Zhihao Wei[3], Hilary A. Dugan[4], Paul C. Hanson[4], and Vipin Kumar[1]

[1]University of Minnesota, Department of Computer Science and Engineering, Minneapolis, 55455, USA
[2]Virginia Tech, Department of Computer Science, Blacksburg, 24060, USA
[3]Beijing University of Technology, Department of Information and Communication Engineering department, Beijing, 100124, China
[4]Center for Limnology, University of Wisconsin-Madison, Madison, WI, 53706, USA
[*]corresponding author: Ankush Khandelwal (khand035@umn.edu)

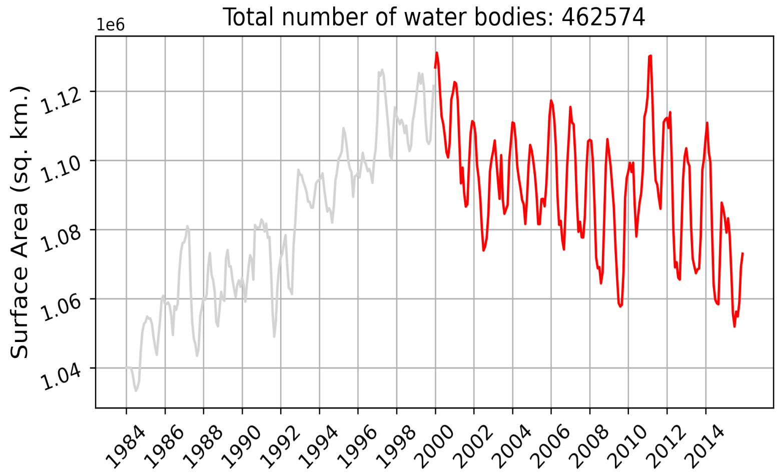

Figure S 1. Aggregate surface area dynamics of 462574 lakes in ReaLSAT with reliable surface extent variations.

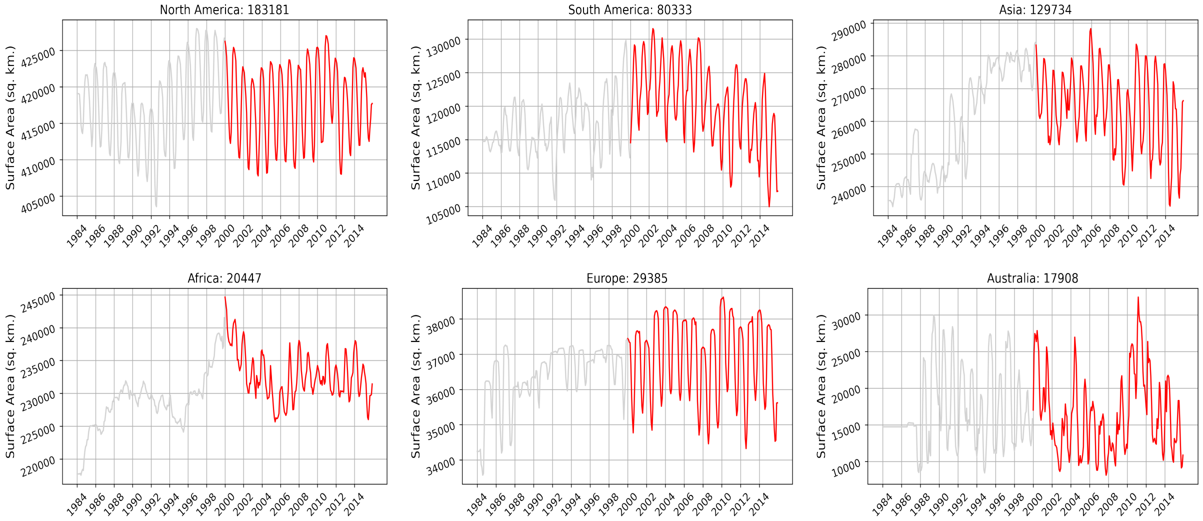

Figure S 2. Aggregate surface area dynamics of all reliable lakes across different continents.

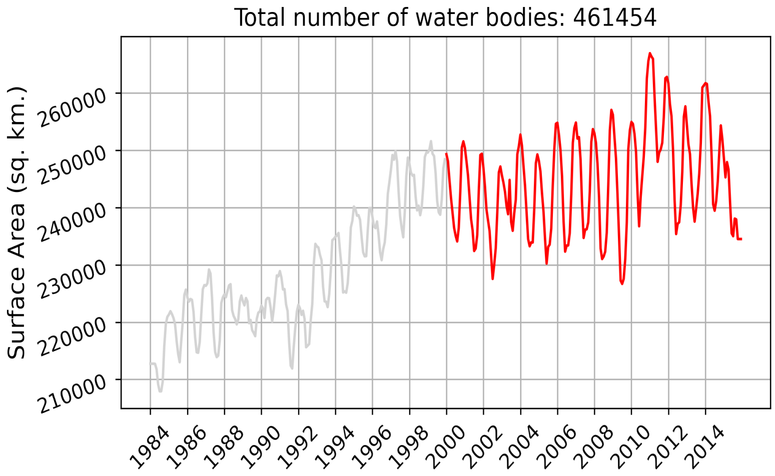

Figure S 3. Aggregate surface area dynamics of 461454 small lakes (area less than 100 sq. kms.) in ReaLSAT with reliable surface extent variations.

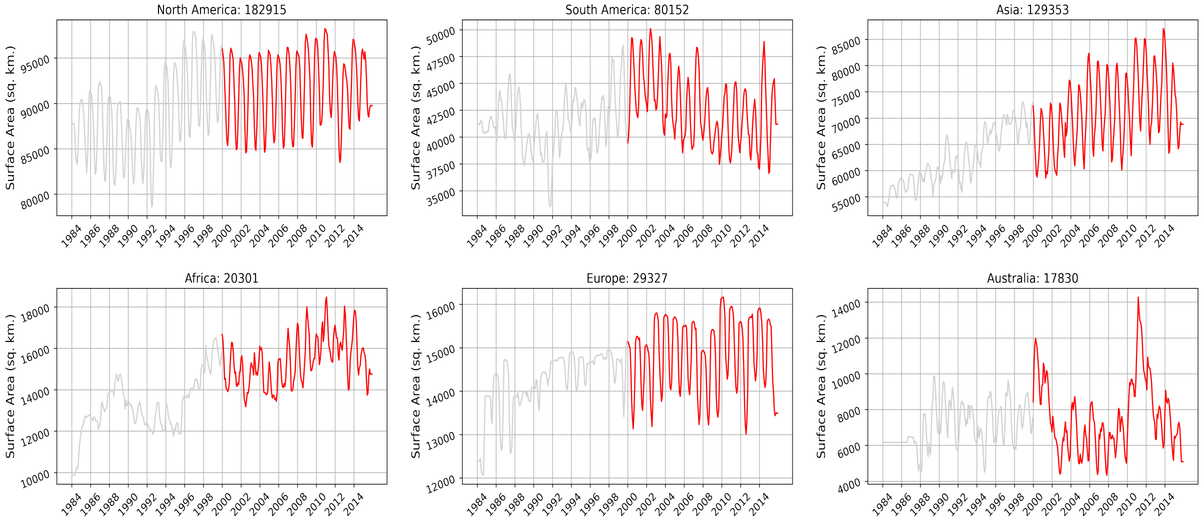

Figure S 4. Aggregate surface area dynamics of small lakes (area less than 100 sq. kms.) across different continents.

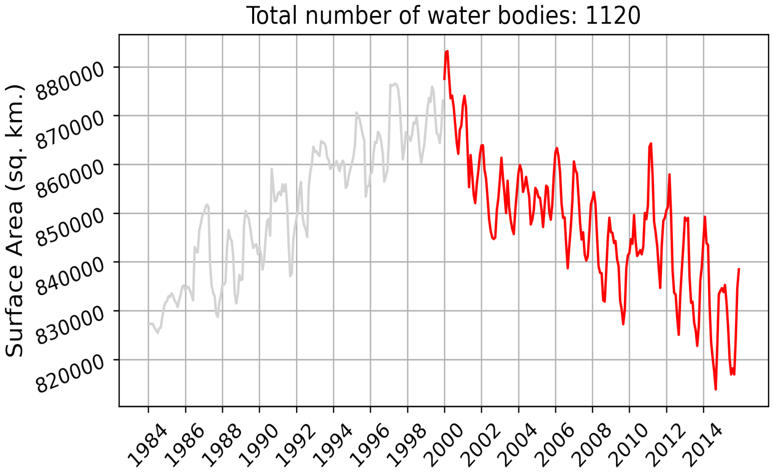

Figure S 5. Aggregate surface area dynamics of 1120 large lakes (area greater than 100 sq. kms.) in ReaLSAT with reliable surface extent variations.

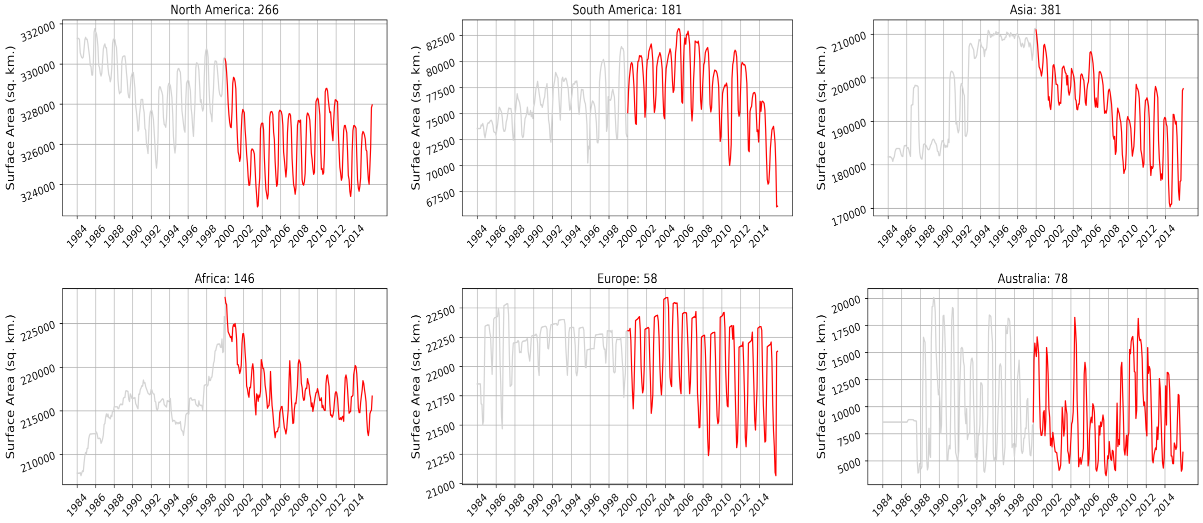

Figure S 6. Aggregate surface area dynamics of large lakes (area greater than 100 sq. kms.) across different continents.

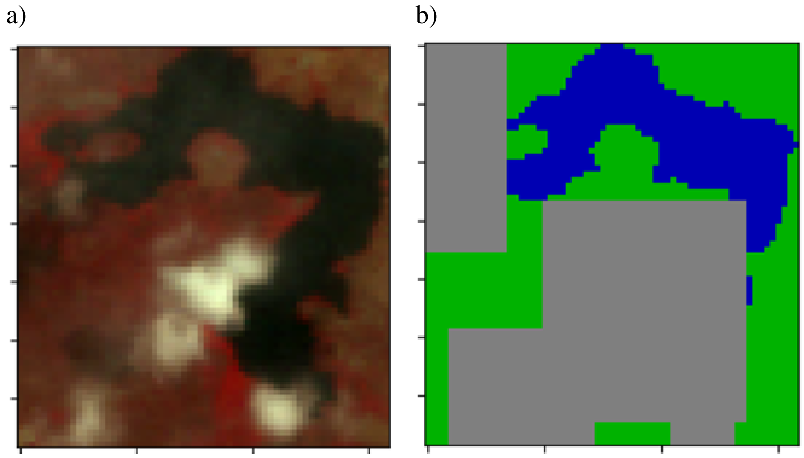

Figure S 7. An illustrative example of a reference map. (a) False Color Composite (using NIR, Red and Green band) image from LANDSAT-5. (b) Reference map created using the semi-automatic process. Green represents land, blue represents water, and gray represents pixels with no reference labels.

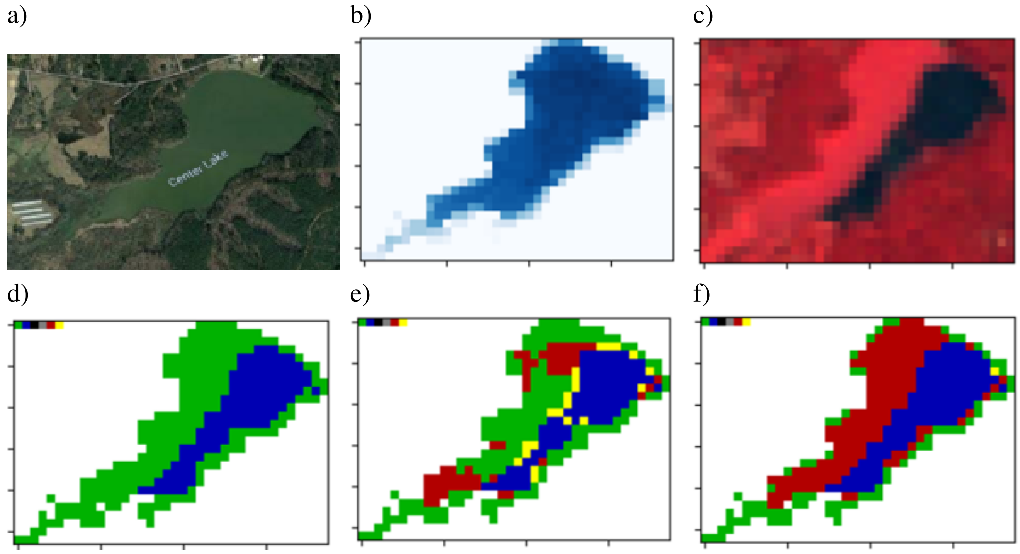

Figure S 8. An illustrative example of the impact of algae on the performance of the ORBIT approach. a) Google Earth imagery of Center Lake, Texas. b) Fraction map created from the GSW dataset that represents percentage of months a given pixel is labeled as water. The color range changes from white to light blue to dark blue represents an increasing fraction from 0 to 100. A bimodal distribution of fraction values (either low or high) reveals high confidence in lake persistence. c) False color composite image on Oct 22, 2008 from LANDSAT-5. Algae has similar reflectance as surrounding vegetation. Color legend for extent maps - water (blue), land (green), gray (missing data), red (water false positives), and yellow (water false negatives). d) Reference extent map creating using Landsat-5 image on Oct 22, 2008. e) GSW extent map. f) ReaLSAT extent map..

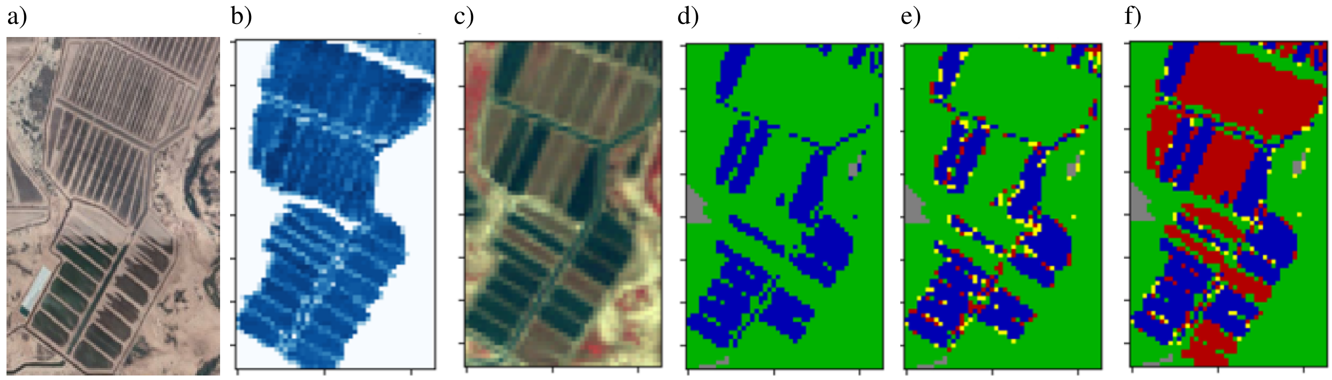

Figure S 9. An illustrative example that highlights the performance issues for agricultural ponds. a) Google Earth imagery of Mexican agricultural ponds on Mar 6, 2021. b) Fraction map created from the GSW dataset that represents percentage of months a given pixel is labeled as water. The color range changes from white to light blue to dark blue represents an increasing fraction from 0 to 100. A bimodal distribution of fraction values (either low or high) reveals high confidence in pond persistence. c) Multi-spectral Landsat-5 image from Oct 8, 2009. Color legend for extent maps - water (blue), land (green), gray (missing data), red (water false positives), and yellow (water false negatives). d) Reference extent map creating using Landsat-5 image on Oct 8, 2009. e) GSW extent map. f) ReaLSAT extent map.

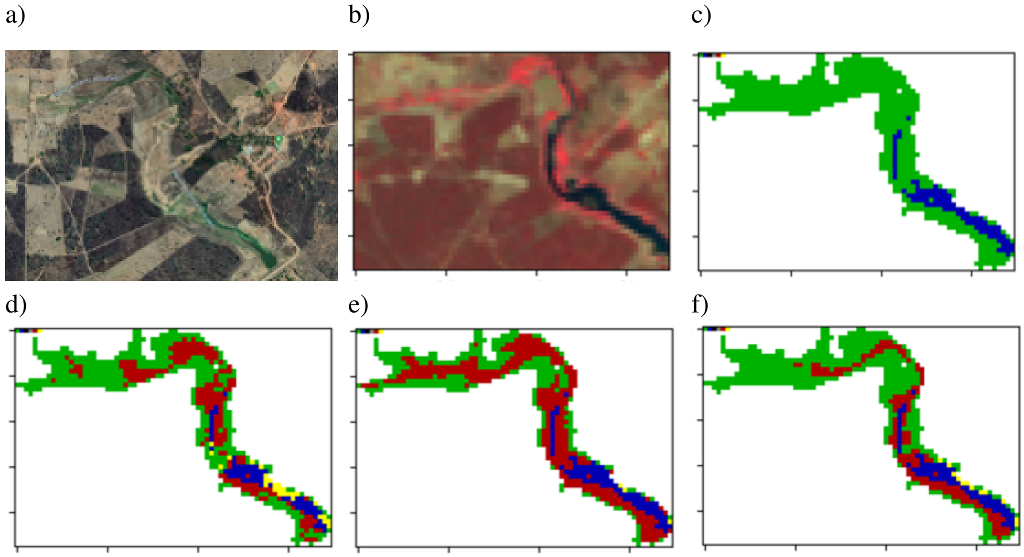

Figure S 10. An illustrative example that highlights the impact of weighting factor on the performance of the ORBIT approach. a) Google Earth imagery from Sep 16, 2020 for a small reservoir in eastern Brazil. b) Multi-spectral Landsat-5 image from Jan 22, 2007. Color legend for extent maps - water (blue), land (green), gray (missing data), red (water false positives), and yellow (water false negatives). c) Reference extent map creating using LANDAT-5 image on Jan 22, 2007. d) GSW extent map. e) ReaLSAT extent map using weight factor of 3. f) ReaLSAT extent map using weight factor of 1.

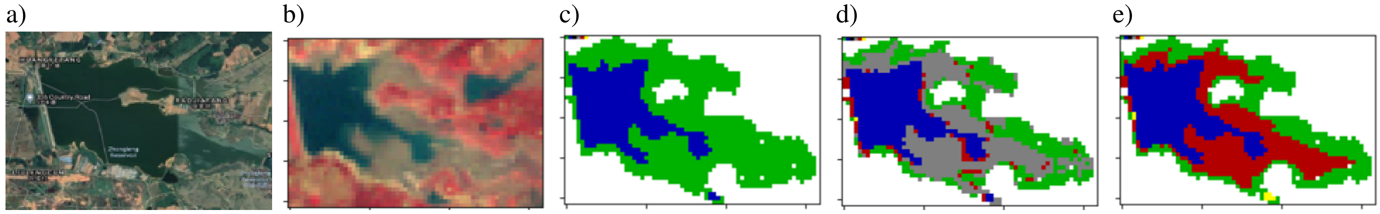

Figure S 11. An illustrative example that highlights the impact of class conditional missing on the performance of the ORBIT approach. a) Google Earth imagery of Zhongleng Reservoir in China on Apr 18, 2018. b) Multi-spectral Landsat-5 image from Nov 2, 2000. Color legend for extent maps - water (blue), land (green), gray (missing data), red (water false positives), and yellow (water false negatives). c) Reference extent map created using Landsat-5 image on Nov 2, 2000. d) GSW extent map. e) ReaLSAT extent map.
